# A Single Nucleotide Polymorphism in *lptG* Increases Tolerance to Bile Salts, Acid, and Staining of Calcofluor-Binding Polysaccharides in *Salmonella enterica* Serovar Typhimurium E40

**DOI:** 10.3389/fmicb.2021.671453

**Published:** 2021-06-02

**Authors:** Taylor A. Wahlig, Eliot Stanton, Jared J. Godfrey, Andrew J. Stasic, Amy C. L. Wong, Charles W. Kaspar

**Affiliations:** ^1^Department of Bacteriology, University of Wisconsin, Madison, WI, United States; ^2^U. S. Food and Drug Administration, Center for Biologics Evaluation and Research, Washington, DC, United States

**Keywords:** *lptG*, LPS, SNP, bile salts, acid tolerance, extracellular polymeric substance, *S. enterica*

## Abstract

The outer membrane of *Salmonella enterica* plays an important role in combating stress encountered in the environment and hosts. The transport and insertion of lipopolysaccharides (LPS) into the outer membrane involves lipopolysaccharide transport proteins (LptA-F) and mutations in the genes encoding for these proteins are often lethal or result in the transport of atypical LPS that can alter stress tolerance in bacteria. During studies of heterogeneity in bile salts tolerance, *S. enterica* serovar Typhimurium E40 was segregated into bile salts tolerant and sensitive cells by screening for growth in TSB with 10% bile salts. An isolate (E40V) with a bile salts MIC >20% was selected for further characterization. Whole-genome sequencing of E40 and E40V using Illumina and PacBio SMRT technologies revealed a non-synonymous single nucleotide polymorphism (SNP) in *lptG*. Leucine at residue 26 in E40 was substituted with proline in E40V. In addition to growth in the presence of 10% bile salts, E40V was susceptible to novobiocin while E40 was not. Transcriptional analysis of E40 and E40V, in the absence of bile salts, revealed significantly greater (*p* < 0.05) levels of transcript in three genes in E40V; *yjbE* (encoding for an extracellular polymeric substance production protein), *yciE* (encoding for a putative stress response protein), and an uncharacterized gene annotated as an acid shock protein precursor (ASPP). No transcripts of genes were present at a greater level in E40 compared to E40V. Corresponding with the greater level of these transcripts, E40V had greater survival at pH 3.35 and staining of Calcofluor-binding polysaccharide (CBPS). To confirm the SNP in *lptG* was associated with these phenotypes, strain E40E was engineered from E40 to encode for the variant form of LptG (L26P). E40E exhibited the same differences in gene transcripts and phenotypes as E40V, including susceptibility to novobiocin, confirming the SNP was responsible for these differences.

## Introduction

*Salmonella enterica* is an important human pathogen that is responsible for an estimated 21 million cases of typhoid fever, 100 million cases of gastroenteritis, and 350,000 deaths annually (Crump et al., 2004; Majowicz et al., 2010). *S. enterica* is associated with a range of animals and usually transmitted to humans by contaminated water and foods (Bäumler, 1997; Groisman and Ochman, 1997; Mahon et al., 1997; Threlfall, 2002; Isaacs et al., 2005; Lan et al., 2009; Newell et al., 2010; Painter et al., 2013; Dechet et al., 2014). Most serovars cause self-limiting gastroenteritis in humans while Typhi strains can cause persistent infections, partly through formation of biofilms within the bile rich gall bladder (Lai et al., 1992; Crawford et al., 2010; Gonzalez-Escobedo et al., 2011; Gonzalez-Escobedo and Gunn, 2013; Gunn et al., 2014). The success of *S. enterica* as a pathogen is due in part to the level of diversity within the species. *S. enterica* contains six subspecies that can be further divided into more than 2,000 serovars (Bäumler, 1997; Groisman and Ochman, 1997; Lan et al., 2009). Within individual *Salmonella* serovars, genetic differences can provide advantages such as antibiotic resistance or antigenic variation, that can enhance *Salmonella* fitness during infection (Piddock et al., 1998; Giraud et al., 1999; Bonifield and Hughes, 2003; Levy et al., 2004; Aldridge et al., 2006; Le et al., 2007; Stewart and Cookson, 2014).

*Salmonella* encounters an array of environmental assaults during transmission and following ingestion, including low pH in the stomach and bile secreted from the gall bladder. These stresses can be mitigated through activation of general stress regulons, RpoS (Hengge-Aronis, 2002; Battesti et al., 2011) and RpoH (Guo and Gross, 2014; Roncarati and Scarlato, 2017), as well as stress specific pathways such as the PhoPQ operon (Foster and Hall, 1990; Spector and Kenyon, 2012), the marRAB operon (Prouty et al., 2004a), and the acrAB operon (Nikaido et al., 2008). The outer membrane (OM), including lipopolysaccharides (LPS), and extracellular polymeric substance (EPS) are central to protection from environmental conditions and signaling pathways (Nikaido, 2003; Flemming and Wingender, 2010; Zhang et al., 2013; Flemming, 2016).

Extracellular polymeric substance is a complex mixture of proteins, polysaccharides, and additional components that form a protective layer around the cell and promote biofilm formation. In contrast to the multicomponent EPS, *Salmonella* LPS is a defined extracellular structure comprised of membrane embedded Lipid A, core oligosaccharides, and the O-antigen polysaccharide. The production and export of LPS is important for virulence (Zhang et al., 2013). Rough mutants, which produce LPS without O-antigen, have reduced virulence, but can yield persisters since O-antigen elicits a strong immune response (Chart et al., 1991; Freudenberg et al., 2008).

**FIGURE 1:**
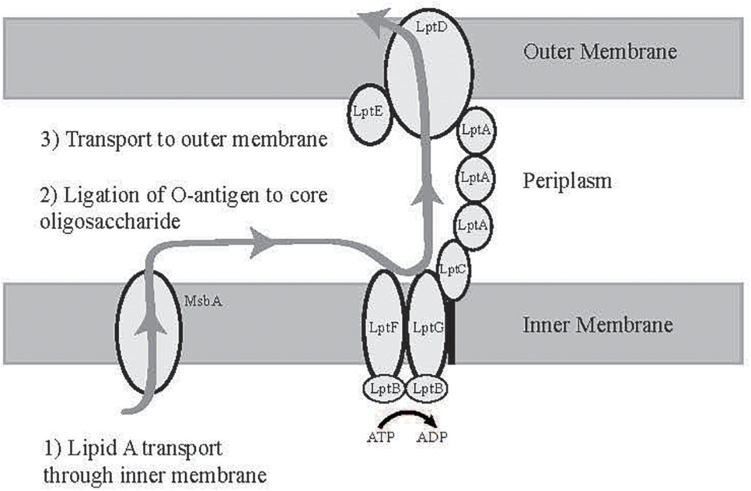
Diagram of LPS transport to the outer membrane. Lipid A is synthesized at the cytoplasmic face of the inner membrane and transported to the periplasm by MsbA, ligated to O-antigen, and then transported to the outer membrane by the Lipopolysaccharide Transport Proteins (LptA-F) (Whitfield and Trent, 2014).

As shown in Figure 1, lipid A is ligated to the core oligosaccharide in the cytosol and transported to the periplasmic face of the inner membrane (IM) by the ABC transporter MsbA (Kalynych et al., 2014; Whitfield and Trent, 2014; Okuda et al., 2016). Independently, O-antigen monomers are transported through the inner membrane and polymerized into complete O-antigen polysaccharides, which are then ligated to the core oligosaccharide in the periplasmic space. Fully assembled LPS is transferred through the periplasmic space and the OM by the lipopolysaccharide transport proteins (LptA-G). LptG and LptF form a transmembrane complex in the IM associated with a homodimer of the ATP-binding protein LptB. LPS that is anchored to the periplasmic face of the IM enters the LptGF transmembrane domain and is shuttled using ATP hydrolysis through the periplasmic space by LptA and LptC, which complex with LptFG and form a link to the OM LptDE complex. LPS is then transferred through the large beta barrel structure within LptD to reach the OM (Sperandeo et al., 2007; Ruiz et al., 2008; Narita and Tokuda, 2009; Dong et al., 2014, 2017; Okuda et al., 2016; Luo et al., 2017; Sherman et al., 2018).

*Salmonella* serovars were screened for the ability to grow in TSB with 10% bile salts. This screen identified a *S. enterica* Typhimurium E40 variant (E40V) that was significantly more tolerant to bile salts (MIC >20%) than a majority of the cells in the E40 culture as well as other serovars. Analysis of E40 and E40V in the absence of bile salts found a greater steady state level of transcripts in three genes in E40V: *yjbE* (an extracellular polymeric substance production gene), *yciE* (a putative stress response protein), and Acid Shock Protein Precursor. The greater level of transcripts for these genes was associated with phenotypes that matched their predicted functions. DNA sequencing and site directed mutagenesis confirmed the SNP in *lptG* was involved in the difference in the level of three transcripts and novobiocin sensitivity.

## Materials and Methods

### Bacterial Strains, Growth, and Storage

The strains and plasmids used in this study are listed in Table 1. Frozen stock cultures were stored in Luria Bertani (LB) broth with 15% glycerol at −70°C. Working stock cultures were cultured on LB agar (Becton Dickinson, Sparks, MD, United States), incubated at 37°C for 20–24 h, and maintained at 4°C for up to 1 month. Overnight cultures were grown from a single colony in 5 ml of tryptic soy broth (TSB; Becton Dickinson) at 37°C for 20–24 h. For bile salts tolerance screening, TSB with 10% bile salts was inoculated with inocula ranging from10^3^ to 10^5^ CFU/ml. For survival and regrowth experiments, TSB with 10% bile salts was inoculated with approximately 10^6^ CFU/ml and numbers of CFU/ml determined periodically during static incubation at 37°C.

**TABLE 1:** Strains and plasmids used in this study.

**Strains and plasmids**	**Characteristics**	**Source**
*S. enterica* Typhimurium E40	chicken isolate	Glass, UW-Madison
*S. enterica* Typhimurium E40V	E40 *lptG* variant	this study
*S. enterica* Typhimurium E40E^a^	E40 with engineered LtpG L26P	this study
*S. enterica* Typhimurium M-090001A-1	peanut butter outbreak	MN Department of Health
*S. enterica* Tennessee 4539H	dry milk outbreak	Food and Drug Administration
*S. enterica* Enteritidis FRIK671	human isolate	Austria
*E. coli* DH5 alpha	cloning host	Kaspar, UW-Madison
pCas9-CR4	inducible Cas9 lamda red vector	Addgene^b^
pKDsgRNA-p15	sgRNA targeting pCas9-CR4	Addgene
pKDsgRNA-*lptG*	sgRNA targeting E40 *lptG*	this study

*^*a*^Strain E40 engineered using No-SCAR editing system (92, 93). ^*b*^Addgene, www.addgene.org.*

### Isolation of Bile Salts Tolerant and Sensitive Cells

**FIGURE 2:**
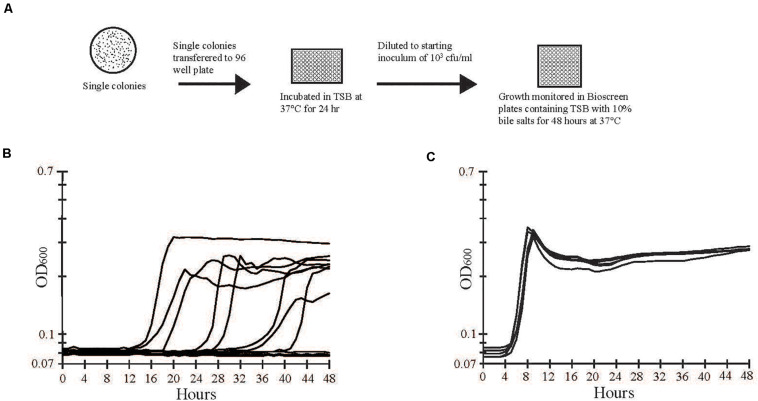
Separation and screening of bile salts tolerant and sensitive strains. **(A)** A stock culture of strain E40 was grown in TSB and then diluted and plated on LB agar to obtain single colonies. Individual colonies were inoculated into separate wells of a 96-well plate containing TSB and incubated overnight at 37°C. Growth from each well was diluted to approximately 10^3^ CFU/ml and used to inoculate separate wells of a Bioscreen plate containing TSB with 10% bile salts. Growth was monitored by optical density at 600 nm during incubation at 37°C for 48 h. **(B)** A representative sample of optical density measurements from growth -positive and -negative wells are shown. Growth varied in the length of lag phase, slope of exponential phase, and maximum optical density. **(C)** Cells from growth-positive wells in **(B)** were transferred to wells of a new plate with TSB and 10% bile salts. The lag, exponential, and stationary phases of growth were consistent among bile salts tolerant cells.

Bile salts (Sigma Aldrich, St. Louis, MO, United States) were an equal mixture of sodium cholate and sodium deoxycholate. Bile salts added to TSB were sterilized by filtration (0.22 μm-pore filter). A diagram of the isolation of bile salts tolerant cells is shown in Figures 2A,B. Overnight cultures were diluted in 0.01 M phosphate-buffered saline (PBS; pH 7.4), plated on tryptic soy agar (TSA; Becton Dickinson), and incubated at 37°C for 24 h. Individual colonies (288 per trial) were inoculated in 200 μl of TSB in 96-well plates (Becton Dickinson) and incubated at 37°C for 24 h. Cells from each well were diluted and inoculated into TSB with 10% bile salts at a final concentration of approximately 10^3^ CFU/ml (Figure 2A). Plates were incubated at 37°C for 48 h, and growth was monitored by measuring optical density (OD_600nm_) every hour using a Bioscreen C (Growth Curves USA, Piscataway, NJ, United States). Wells exhibiting a change in OD_600nm_ greater than 0.1 units from the baseline reading were considered positive for growth. Cells from growth-positive and growth-negative wells were recovered and stored at −70°C.

### Survival in Bile Salts

Survival in TSB with 10% bile salts was monitored by inoculating approximately 10^6^ cells of E40 or E40V and determining the number of CFU/ml with LB agar plates after 3, 16, and 20 h of incubation at 37°C.

### Antibiotic Susceptibility Testing

Cells from overnight cultures were spread with a swab on LB agar plates to create lawns. Discs of Whatman filter paper containing ampicillin (10 μg/disc), novobiocin (0.5 μg/disc), or polymyxin B (1 μg/disc) were placed on the swabbed plates. Plates were incubated at 37°C for 18 h, and zones of growth inhibition were measured.

### Pulsed-Field Gel Electrophoresis

Exponential phase cells were harvested, washed with 1 M NaCl and 10 mM Tris–HCl, and suspended in a 1% low melting point agarose (Gibco Brl, Gaithersburg, MD, United States). Cells were lysed and washed as previously described (Tamplin et al., 1996; Buchrieser et al., 1997). Genomic DNA in agarose plugs was digested with XbaI (New England Biolabs, Ipswich, MA, United States) at 37°C for 20 h. High molecular weight fragments were resolved using a 1.3% ultrapure agarose gel (J. T. Baker, Philipsburg, PA, United States) in a CHEF-DR II pulsed-field system (Bio-Rad Laboratories, Richmond, CA, United States). Electrophoretic parameters were 6 V/cm for 22 h at 15°C with switching times from 5 to 50 s. Gels were stained overnight in ethidium bromide and visualized using UV light.

### DNA Sequencing and Analysis

E40 and E40V were sequenced by Illumina and PacBio SMRT sequencing. For Illumina sequencing, E40 and E40V cultures were individually inoculated into LB broth directly from frozen stock cultures. Following incubation overnight at 37°C, cells were harvested by centrifugation. DNA was prepared using MasterPure Complete DNA and RNA Purification Kit (Epicentre, Madison, WI, United States). RNA was removed by the addition of RNase A (Thermo Fisher Scientific, Waltham, MA, United States) and incubation for 30 min at 37°C. The manufacturer’s protocol was modified to include a step to precipitate DNA by overnight incubation in 70% ethanol at −20°C. DNA was submitted to the University of Wisconsin-Madison Biotechnology Center for sequencing. DNA concentration was verified using the Qubit^®^ dsDNA HS Assay Kit (Life Technologies, Grand Island, NY, United States) and prepared with a TruSeq Nano DNA LT Library Prep Kit (Illumina Inc., San Diego, CA, United States) with minor modifications. Samples were sheared using a Covaris M220 Ultrasonicator (Covaris Inc., Woburn, MA, United States), and size selected for an average insert size of 550 bp using SPRI bead-based size exclusion. The quality and quantity of the finished libraries were assessed using an Agilent High Sensitivity DNA kit and Qubit^®^ dsDNA HS Assay Kit, respectively. Libraries were standardized to 2 nM and paired-end 250 bp sequencing was performed using an Illumina MiSeq Sequencer and a MiSeq 500 bp (v2) sequencing cartridge. Images were analyzed using the standard Illumina Pipeline, version 1.8.2.

Genomic DNA for PacBio sequencing was purified from overnight cultures that were harvested by centrifugation and washed four times with sterile 10% glycerol. DNA from washed cell pellets was purified by CTAB DNA extraction (William et al., 2004). Standard Pacific Biosciences large insert library preparation was performed at the UW-Milwaukee Great Lakes Genomics Center. DNA was fragmented to approximately 20 kb using Covaris G tubes (Covaris, Woburn, MA, United States). Fragmented DNA was enzymatically repaired and ligated to a PacBio adapter to form the SMRTbell Template. Templates larger than 10 kb were size selected by BluePippin (Sage Science, Beverly, MA, United States). Templates were annealed to sequencing primer, bound to polymerase (P6) and then PacBio Mag – Beads and SMRT cell, and sequenced using C4 chemistry.

Genome assemblies were created from PacBio reads using SPAdes (Bankevich et al., 2012). The resulting contigs underwent iterative improvement using Pilon (Walker et al., 2014) utilizing short-read Illumina data that was corrected using the BayesHammer module (Nikolenko et al., 2013) included in SPAdes 3.11.1. Annotations were generated using Rapid Annotation Using Subsystem Technology (RAST) (Aziz et al., 2008; Overbeek et al., 2014; Brettin et al., 2015).

Comparisons between E40 and E40V were performed using Mauve (Darling et al., 2010), BLAST (Madden, 2002), MUSCLE (Edgar, 2004), and IGV (Robinson et al., 2012). Regions containing differences in the E40 and E40V assemblies were amplified by PCR and sequenced by Sanger sequencing (Sanger et al., 1977) at the UW-Madison Biotechnology Center. Primers used are shown in Supplementary Table 1.

### mRNA Analysis

RNA was purified using standard Trizol purification (Rio et al., 2010) from cells grown to OD_600nm_ = 0.3 in TSB (pH 7.3). Total RNA was submitted to the University of Wisconsin-Madison Biotechnology Center and verified for purity and integrity via the NanoDrop2000 Spectrophotometer and Agilent 2100 BioAnalyzer, respectively. Samples that met the Illumina sample input guidelines were prepared according to the TruSeq^®^ RNA Sample Preparation Guide (Rev. C, May 2012). For each sample, 2 μg of total RNA underwent ribosomal RNA (rRNA) reduction using the EpiCentre Ribo-Zero^TM^ rRNA Removal (Gram-Negative Bacteria) kit (EpiCentre Inc., Madison, WI, United States). The rRNA-depleted samples were purified by ethanol precipitation and quantified on the Qubit fluorimeter with the Qubit^®^ RNA Assay Kit (Invitrogen, Carlsbad, CA, United States). rRNA-depleted samples (60 ng) were fragmented, using divalent cations under elevated temperature, and immediately reverse transcribed into double-stranded (ds) cDNA using SuperScript II Reverse Transcriptase (Invitrogen) and random primers. The ds cDNA was purified with paramagnetic beads, end-repaired by T4 DNA polymerase and Klenow DNA Polymerase, and phosphorylated by T4 polynucleotide kinase. The blunt ended cDNA was purified with paramagnetic beads and then incubated with Klenow DNA Polymerase to add an “A” base (Adenine) to the 3′ end of the blunt phosphorylated DNA fragments. DNA fragments were ligated to Illumina adapters, which have a single “T” base (Thymine) overhang at their 3′end. The adapter-ligated products were again purified with paramagnetic beads. Products of the ligation were PCR-amplified with Phusion^TM^ DNA Polymerase using Illumina’s genomic DNA primer set and purified with paramagnetic beads. The quality and quantity of the DNA were assessed using an Agilent DNA 1000 series chip assay and Invitrogen’s RiboGreen detection dye. Cluster generation and sequencing were performed on the Illumina cBot and HiSeq2000 following manufacturer’s instructions. Reads were mapped to the strain E40V genome assembly using Rsubread (Liao et al., 2013) and statistical comparisons were performed using the nbinomWaldTest performed by DESeq2 (Love et al., 2014). Alignment statistics are shown in Supplementary Table 2, and raw expression analysis is shown in Supplementary Table 3. Differential expression was considered significant with a Benjamini-Hochberg adjusted *p* value <0.05 (Benjamini and Hochberg, 1995). Differential expression was confirmed by reverse transcriptase quantitative PCR (RT-qPCR), using independently purified RNA samples. RT-qPCR analysis was performed using a Bio-Rad CFX Connect Real-Time System. *recA* and *rpoB* were used as reference genes, and primers (Supplementary Table 1) were designed using primer3 (Thornton and Basu, 2011; Untergasser et al., 2012). RT-qPCR data were analyzed (Pfaffl, 2001) using two reference genes.

### Acid Challenge

TSB (pH 7.8) was inoculated 1:500 from overnight cultures. Cultures were grown at 37°C with shaking (100 rpm) to OD_600nm_ = 0.2. Cells were diluted and inoculated in LB pH 3.0, which resulted in a final challenge pH of 3.35. Cultures were incubated statically at 37°C for 6 h. Samples were serially diluted in 0.1% peptone (pH 7.0), plated on LB agar, incubated overnight at 37°C, and the number of colony-forming units enumerated.

### Detection of CBPS

Tryptic Soy Agar (MP Biomedicals, Solon, OH, United States) plates containing 1% calcofluor white were spotted with 2 μl of overnight culture. Plates were incubated at 37°C for 3 days. Calcofluor incorporation was visualized with long-wave UV light and images recorded using an Olympus E-PM1 camera (Richter et al., 2014; Hawkins et al., 2017). Staining was quantified using the Fiji distribution of ImageJ (Schindelin et al., 2012; Schneider et al., 2012).

### Extraction and LPS Examination

Cells from overnight cultures grown in LB were normalized to OD_600nm_ = 1.0 and collected by centrifugation. LPS was extracted with hot aqueous-phenol as described previously (Davis Jr. and Goldberg, 2012). Extracted LPS was separated on a Tris-Tricine gel (Lesse et al., 1990) and visualized using a Pierce Silver Stain kit (Thermo Fisher Scientific, Waltham, MA, United States). Quantification of band intensity was performed using the Fiji distribution of ImageJ (Schindelin et al., 2012; Schneider et al., 2012).

### Engineering of E40E

Strain E40E was engineered using No-SCAR genome editing (Reisch and Prather, 2015). pKDsgRNA:*lptG* was generated using CPEC (Reisch and Prather, 2017). The repair template (Supplementary Table 1) was designed to introduce the desired SNP as well as an additional synonymous SNP to reduce cas9 targeting (Table 2). This change also created a Bsr1 (NEB) cut site, which was used to identify recombinants. One out of 72 recombinants screened was positive by Bsr1 digestion and was confirmed to have the desired modification by Sanger sequencing (Sanger et al., 1977).

**TABLE 2:** DNA and amino acid sequence of positions 24–28 in *lptG* and LptG of strains E40, E40V, and E40E.

		**LptG position**				
**Strain**		**24**	**25**	**26**	**27**	**28**
E40	amino acids	F	M	L	V	S
	nucleotides	TTC	ATG	CTG	GTG	TCG
E40V	amino acids	F	M	P	V	S
	nucleotides	TTC	ATG	CCG	GTG	TCG
E40E	amino acids	F	M	P	V	S
	nucleotides	TTC	ATG	CCA	GTG	TCG

### Statistical Analysis

The number of independent biological replicates conducted is presented within individual figure legends. Standard error is shown. Alignments of RNA sequencing reads were created using Rsubread (Liao et al., 2013) and statistical comparisons were performed using DESeq2 using the nbinomWaldTest (Love et al., 2014). For RNAseq experiments, Benjamini-Hochberg adjusted *p* values <0.05 were considered significant. RT-qPCR data were analyzed (Pfaffl, 2001) with two reference genes. *P* values were determined using the student’s *t* test (2-tailed).

## Results

### Heterogeneity in *S. enterica* Growth in TSB With 10% Bile Salts

Two hundred and eighty-eight colonies from *S.* Typhimurium culture E40 were propagated overnight, diluted to approximately 10^3^ CFU/ml, and inoculated in TSB with 10% bile salts (Figure 2A). Growth was monitored by optical density (OD_600nm_) of individual wells over 48 h. Fifty six percent of the wells had growth that varied in the length of lag phase, slope of exponential phase, and maximal optical density achieved (Figure 2B). Cells from growth-positive wells retained their ability to grow in the presence of 10% bile salts but had a shorter lag phase and more uniform growth than the initial growth curves (Figure 2C). The frequencies of growth-positive colonies from *S. enterica* Typhimurium strain M-09-0001A-1 (42%) and *S. enterica* Enteriditis strain FRIK671 (64%), were similar to *S. enterica* Typhimurium E40 (56%). In contrast, *S. enterica* Tennessee strain 4539H had a nearly 100-fold lower frequency of growth positive colonies (0.7%). In comparison to *S. enterica* Typhimurium strain E40 (MIC 8%), strain E40V had an especially high bile salts MIC (>20%), which was the highest concentration of bile salts that could be dissolved in TSB without formation of precipitate.

### Strains E40 and E40V Differ by a Single Nucleotide Polymorphism (SNP)

Strains E40 and E40V were both serotyped and confirmed as serovar Typhimurium. Additionally, pulsed-field gel electrophoresis of XbaI digests (Supplementary Figure 1) did not show any discernible chromosome differences between E40 and E40V. Results from serotyping and pulsed-field gel electrophoresis ruled out that the culture was a mixture of different serovars or Typhimurium strains. To determine if genetic differences accounted for the discrepancy in bile salts tolerance, paired-end Illumina and PacBio sequencing of E40 and E40V was performed. Illumina sequencing resulted in 114× and 113× coverage of E40 and E40V, respectively (Supplementary Table 4A). PacBio sequencing resulted in average read lengths of 8,020 and 8,908 bp, with coverage of 189× and 164× for E40 and E40V, respectively (Supplementary Table 4B). Assembly of PacBio reads for E40 and E40V both resulted in two contigs, representing the chromosome and one plasmid. These contigs were polished using paired-end Illumina reads (Supplementary Table 4C). The E40 chromosome assembly (4,890,368 bp) was 30 bp longer than the E40V chromosome assembly (4,890,338 bp). The plasmid assemblies for both E40 and E40V were identical (94,009 bp). The E40 (NCBI accession number CP038432-CP038433) and E40V (NCBI accession number CP038434-CP038435) chromosome and plasmid assemblies were compared using a combination of Mauve (Darling et al., 2010), BLAST (Madden, 2002), MUSCLE (Edgar, 2004), and IGV (Robinson et al., 2012). Six regions were identified outside of rRNA and tRNA regions with nucleotide differences, ranging from individual SNPs to a 4-codon deletion. Sanger sequencing was used to reexamine the putative differences. Five of the six differences were not confirmed by Sanger sequencing and were considered assembly errors (Supplementary Table 5). These assembly errors accounted for 16 bp of the 30 bp difference between the E40 and E40V chromosome assemblies, and the remaining difference in assembly length was attributed to putative assembly errors in rRNA and tRNA regions. Analysis of the assembly errors revealed that a majority were either in genes that had homologs elsewhere in the genome or were in repetitive regions. The remaining difference was a non-synonymous SNP in the LPS transport gene *lptG* that changed amino acid 26 from a leucine to a proline (p.leu26pro) (Table 2). Analysis of *Salmonella* genomes in the National Center for Biotechnology Information (NCBI) database did not find a match to the E40V *lptG* sequence.

### E40 and E40V Were Present in the Parent Culture

To assess if the SNP found in E40V was present in the parent culture prior to exposure to 10% bile salts, Illumina reads used for transcriptional analysis were analyzed for the presence of the SNP. Reads (726) from E40 replicates were identified that aligned to *lptG*. Two of these reads, each from separate replicates, contained the E40V SNP, indicating the E40V variant was present before exposure to bile salts.

### LtpG (p.leu26pro) Was Responsible for the Observed Phenotypic Differences

**FIGURE 3:**
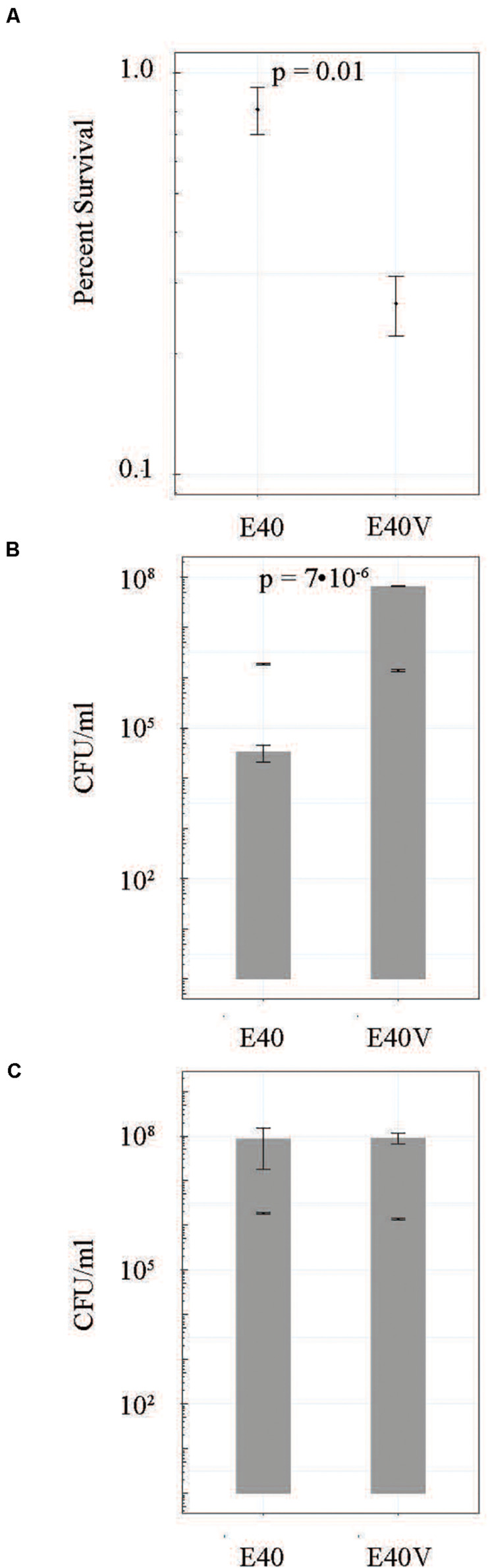
E40 and E40V survival and growth in TSB with 10% bile salts when inoculated at approximately 10^6^ CFU/ml. **(A)** Cells were incubated at 37°C for 3 h and the percent survival was calculated from CFU/ml determinations. **(B)** CFU/ml of E40 and E40V after 16 h of incubation. The bars represent CFU/ml following inoculation. **(C)** CFU/ml after 20 h of incubation. The bars represent CFU/ml following inoculation. *N* = 3 for all time points. Error bars are the standard error of the mean.

To determine if the SNP in *lptG* was responsible for the observed difference in bile salts tolerance, strain E40 was engineered to create strain E40E. Strain E40E encoded for LptG with a proline at position 26, matching the E40V LptG sequence (Table 2). Similar to E40V, strain E40E grew in TSB with 10% bile salts while E40 did not when inoculations ranged from 10^3^ to 10^5^ CFU/ml. We hypothesized that LtpG (p.leu26pro) would increase viability during initial exposure to bile salts; however, the survival of E40V (0.27 ± 0.05%) was significantly lower (p = 0.01) after 3 h in TSB with 10% bile salts than E40 (0.81 ± 0.11%) (Figure 3A). After 16 hours in TSB with 10% bile salts, E40V (6.6 × 10^7^ CFU/ml) had significantly more growth (*p* = 7 × 10^–6^) than E40 (3.3 × 10^4^ CFU/ml) (Figure 2B). At 20 h, E40 and E40V had equivalent numbers of CFU/ml when inoculated with CFU/ml (Figure 3C). Adaptive responses to bile salts can increase resistance to antibiotics (Prouty et al., 2004a) and differences in susceptibility to hydrophobic antibiotics indicate differences in the outer membrane composition or LPS modifications (Delcour, 2010). The hydrophobic antibiotic novobiocin created a zone of inhibition in E40V (12.6 ± 0.5 mm); whereas there was no zone of inhibition with E40 (*p* < 0.01) (Table 3). Like E40V, the engineered strain E40E was inhibited by novobiocin (11.1 ± 0.3 mm). Polymyxin B and ampicillin were also inhibitory but the zones of inhibition were not significantly different between strains.

**TABLE 3:** Zones of inhibition of *S. enterica* strains E40, E40V and E40E with the following antibiotics: novobiocin (1.5 μg/disc), polymyxin B (3 μg/disc), and ampicillin (10 μg/disc).

**Strain**	**Novobiocin^a^**	**Polymyxin B**	**Ampicillin**
E40	0.0^b^	12.2 +/−0.3	25.8 +/−0.3
E40V	12.6 +/−0.5	12.9 +/−0.1	26.0 +/−0.8
E40E	11.1 +/−0.3	12.4 +/−0.2	ND^c^

*^*a*^E40 significantly different from E40V and E40E (*p* < 0.01), *n* = 4. ^*b*^Values are in mm +/−the standard error. ^*c*^ND, not determined.*

**FIGURE 4:**
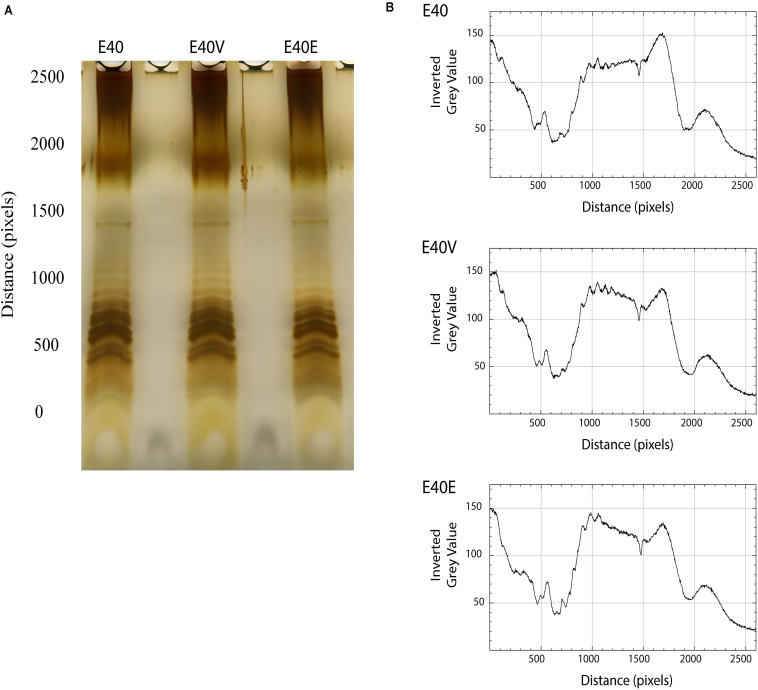
LPS profiles and scans from E40, E40V, and E40E. **(A)** Cells were harvested, the LPS extracted with hot aqueous phenol, separated in a Tris-Tricine gel, and visualized by silver staining. **(B)** The LPS profiles were scanned using ImageJ and bands assessed by gray scale.

Lipopolysaccharides was extracted and visualized by electrophoresis and silver staining to determine if LptG p.leu26pro affected the makeup and quantities of LPS components. In general, the LPS profiles were similar although quantification of the gray scale values of the imaged gel indicated minor differences between strain E40 and strains E40V and E40E. From pixels 1,000–15,00, strains E40E and E40V had peaks and a declining plateau with gray values ranging from 140 to 120 while the peaks and plateau from strain E40 remained nearly constant with gray values around 120 (Figure 4).

### Steady State Levels of *yjbE*, *yciE*, and Acid Shock Protein Precursor (ASPP) Transcripts Were Greater in E40V and E40E

To determine if there were transcriptional differences that contributed to the difference in bile salts tolerance, RNAseq was used to examine exponential-phase cultures of E40 and E40V grown in TSB with no bile salts. Analysis was conducted in the absence of bile salts since the initial separation of E40 and E40V (separate colonies Figure 2A) was conducted in the absence of bile salts. Three genes were identified as differentially expressed in E40 and E40V (Table 4). *yjbE* (involved in EPS production), *yciE* (a putative stress response gene), and ASPP (promotes acid tolerance) were expressed at log_2_ values of 2.0, 3.0, and 1.2 greater in E40V than E40, respectively. No transcripts were present at a significantly greater level in E40 compared to E40V. Greater steady state levels of transcripts were confirmed by RT-qPCR using independently extracted RNA samples, with log_2_ values of 2.6, 1.1, and 0.4 for *yjbE*, *yciE*, and ASPP, respectively (Table 4). The role of the *lptG* SNP (p.leu26pro) in the differences in transcript levels of these three genes was confirmed by RT-qPCR using RNA isolated from E40E. Like E40V, there were greater steady state levels of *yjbE*, *yciE*, and ASPP mRNA in E40E compared to E40 with log_2_ values of 1.6, 2.6, and 0.34, respectively (*p* = 1.5×10^–5^, *p* = 6.6×10^–4^, and *p* = 0.1; respectively). These results confirm that the differences in the level of these gene transcripts in E40V were linked to the SNP in *lptG*.

**TABLE 4:** Strain E40V genes with greater steady state levels of mRNA than strain E40.

		**RNAseq**		**RT-qPCR^a^**	
**Gene**	**Function**	**log_2_-fold change^b^**	***p* value^c^**	**log_2_-fold change^b^**	***p* value^c^**
*yjbE*	EPS^d^ production	2.0	4.8 × 10^–7^	2.6	2.6 × 10^–4^
yciE	putative stress response protein	3.0	1.1 × 10^–5^	1.1	1.3 × 10^–3^
ASPP^e^	acid tolerance	1.2	2.8 × 10^–2^	0.4	6.8 × 10^–2^

*^*a*^Reverse transcriptase quantitative PCR. ^*b*^Average log_2_-fold change from three biological replicates, values are levels in E40V compared to E40. ^*c*^Benjamini-Hochberg adjusted (Benjamini and Hochberg, 1995). Calculated using student’s *t*-test. ^*d*^Extracellular polymeric substance. ^*e*^Acid shock protein precursor.*

### LptG (p.leu26pro) Had Greater Acid Tolerance and Calcofluor-Binding Polysaccharides

**FIGURE 5:**
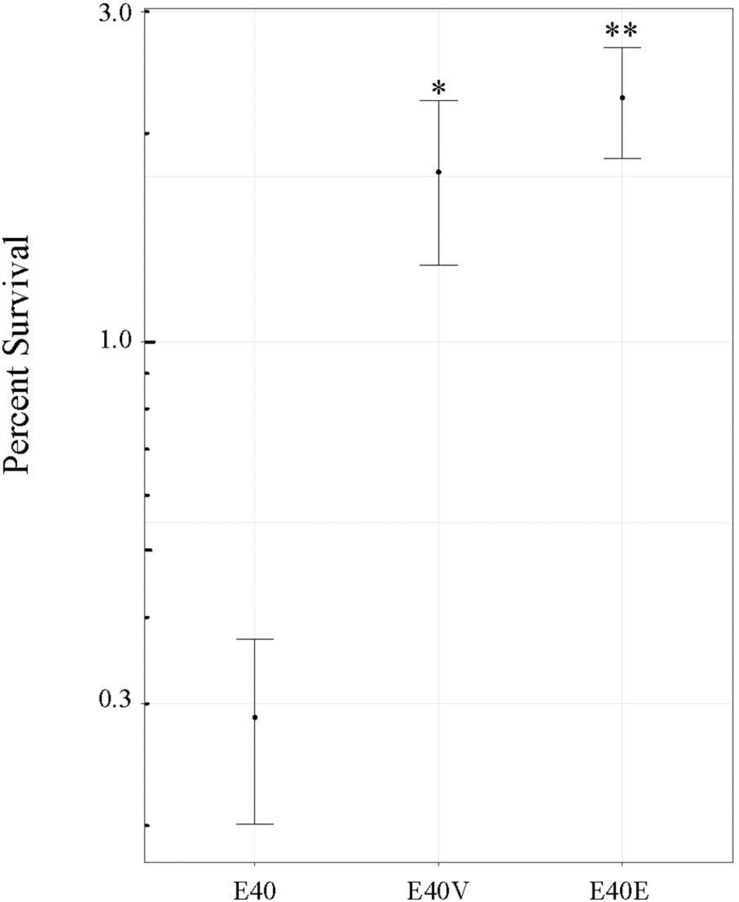
Survival of strains E40, E40V, and E40E at pH 3.0. Strains were inoculated into TSB at pH 3.0 and the number of CFU/ml determined after 6 h of incubation at 37°C. Percent survival was determined using the number of CFU/ml following inoculation and after 6 h of incubation. The average values from four trials are shown. Error bars are the standard error of the means. ^∗^*p* = 0.02 between E40 and E40V. ^∗∗^*p* = 0.003 between E40 and E40E.

**FIGURE 6:**
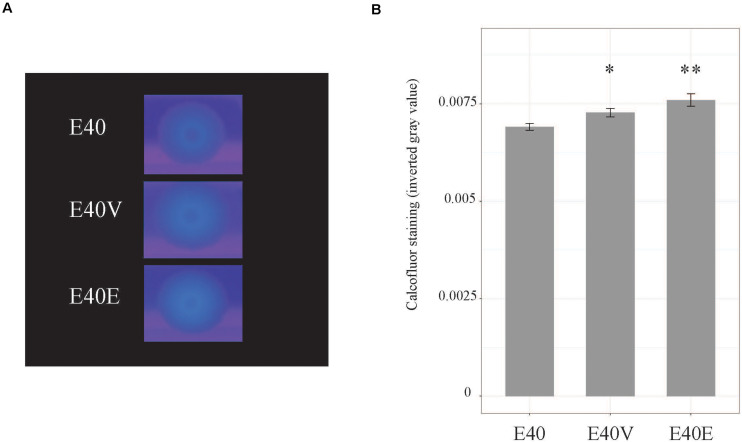
Calcofluor staining of strains E40, E40V, and E40E. **(A)** Cells were spotted on TSA with 1% calcofluor white and incubated at 37°C for 3 days. Calcofluor incorporation was observed by excitation with long-wave UV light. A representative image from four trials is shown. **(B)** Calcofluor staining was measured using ImageJ. Average values from four trials are shown. Error bars are the standard error of the mean. ^∗^*p* = 0.04 between E40 and E40V. ^∗∗^*p* = 0.007 between E40 and E40E.

Based on the differences in steady state level of transcripts, additional phenotypic characterization of E40, E40V, and E40E was performed. Both E40V and E40E showed greater levels of transcript for the gene encoding for the ASPP, which is implicated in acid tolerance (Table 4). Acid challenge (pH 3.35) of E40, E40V, and E40E found that the strains E40V (1.8 ± 0.5%) and E40E (2.25 ± 0.4%) had statistically greater survival (*p* = 0.02 and *p* = 0.003, respectively) than E40 (0.3% ± 0.09%) (Figure 5). Likewise, transcriptional analyses found a greater quantity of *yjbE* transcript; therefore, calcofluor-binding polysaccharides (CBPS) were visualized and quantified using the fluorescent polysaccharide stain calcofluor white. Visual examination showed E40V and E40E colonies had a brighter and wider band of fluorescence than E40 colonies. Measurement of inverted gray value saturation showed values of E40 colonies (0.0069 ± 8.7×10^–5^) were significantly less than E40V (0.0073 ± 1.1×10^–4^) and E40E (0.0076 ± 1.6×10^–4^) (Figure 6). Characterization of E40 and E40V identified three distinguishing phenotypes (growth in TSB with 10% bile salts, acid tolerance, and staining of CBPS). Strain E40E (p.leu26pro) had the same phenotypes as E40V demonstrating that the SNP in *lptG* was linked to the observed phenotypes.

## Discussion

*Salmonella enterica* is a pervasive pathogen that encounters a variety of unfavorable conditions in the environment and hosts. *Salmonella* has multiple stress tolerance pathways, but the outer membrane is an important first line of defense against environmental stressors. This study demonstrated that a SNP (p.leu26pro) in the gene encoding for lipopolysaccharide transport protein G (*lptG*) found in strain E40V increased tolerance to two important stressors (bile salts and acid) that *Salmonella* encounters during human infection. The SNP also increased staining of CBPS by calcofluor. Although strain E40V was able to grow in the presence of 10% bile salts within 48 h, it had lower initial viability in TSB with 10% bile salts compared to E40 (Figure 3A). E40 did not grow in 10% bile salts when inoculated at 10^3^ CFU/ml but did when inoculated at 10^6^ CFU/ml (Figure 3C). The survival curve of E40V was characterized by a die off followed by the outgrowth of a few survivors that is characteristic of the “phoenix phenomenon” that has been described previously (Mellefont et al., 2005). These findings suggest that E40V was not more tolerant to bile salts but rather a few survivors had adapted in a manner that permitted their growth. A possible contributing factor to these observations was E40V had greater steady state level of transcripts from three genes (*yjbE, yciE*, and a gene encoding for acid shock precursor protein) in comparison to strain E40 that corresponded with the observed phenotypes in E40V. The ability of E40V to survive and grow in TSB with 10% bile salts could be driven by the protein products of these genes. The mechanism by which LptG p.leu26pro gives rise to an increase in these three transcripts remains unresolved and is the subject of ongoing research.

While knocking out or knocking down Lpt pathway proteins is deleterious or even lethal to bacteria (Ruiz et al., 2008; Chimalakonda et al., 2011), alterations of Lpt pathway proteins can mitigate other mutations or increase stress tolerance. In *Burkholderia cenocepacia*, LPS is modified with the addition of 4-amino-4-deoxy-L-arabinose (L-Ara4N), which confers resistance to antimicrobial peptides. Knocking out the pathway for L-Ara4N modification generates nonviable cells; however, a SNP in *lptG* restores viability by allowing the export of LPS that does not have the L-Ara4N modification (Hamad et al., 2012). Alteration of LptG in *Pseudomonas aeruginosa* increases susceptibility to some classes of antibiotics, while inducing hypersensitivity to other antibiotics (Harrison et al., 2019). In *Salmonella*, single codon deletions and SNPs in *lptC* and *lptE* increase tolerance to bile salts. Analysis of LPS profiles of the *lptC* and *lptE* mutants found differences in polysaccharide chain length, compared to wild type LPS (Hernández et al., 2012). These studies demonstrate that SNPs in *lpt* genes result in minor changes to Lpt proteins that transport LPS molecules with structural variations that alter the chemical composition or charge of the outer membrane that can impact permeability and stress tolerance.

Analysis of DNA sequence data obtained from multiple sequencing technologies (PacBio, Illumina, and Sanger) identified a single non-synonymous SNP in *lptG* that changed position 26 from a leucine (E40) to a proline (E40V). Phenotypic characterization revealed that this SNP had pleiotropic effects. As has been shown with *lptC* and *lptE*, the mutation in *lptG* found in E40V increased tolerance to bile salts. Additionally, E40V had increased acid tolerance (Figure 5) and staining of calcofluor-binding polysaccharides (CBPS) (Figure 6), when compared to E40. RT-qPCR analysis of RNA from exponential-phase cells showed a greater level of transcripts from three genes (*yciE*, *yjbE*, and ASPP) in E40V and E40E, compared to E40 (Table 4). The predicted function of these genes corresponded with the observed phenotypic differences between E40 and the LptG variants E40V and E40E.

Other studies found that changes in Lpt pathway proteins can alter the LPS present in a strain (Hernández et al., 2012; Bertani et al., 2018). Analysis of LPS from E40, E40V, and E40E using a similar method found only minor differences in the staining intensity of LPS O antigen side chains (Figure 4). Changes to LPS can alter stress tolerance and resistance to antibiotics (Delcour, 2010) similar to the decline in CFU/ml of E40V in bile salts containing medium (Figure 3) and the sensitivity to novobiocin (Table 3). It is unlikely that minor differences in O antigens alter permeability to novobiocin (Sheng et al., 2008); whereas, LPS that is unmodified by PhoPQ allows novobiocin to pass through the membrane (Nobre et al., 2015). It is most likely that LptG (p.leu26pro) resulted in the transport of unmodified or modified LPS that resulted in a membrane permeable to bile salts and novobiocin (Bertani et al., 2018). The sensitivity of E40V and E40E to novobiocin demonstrated the outer membranes differed from E40 and that this difference permitted novobiocin permeability which is typically prevented by PhoPQ-modified LPS (Nobre et al., 2015). The proline in LptG (p.leu26pro) is seven residues away from a series of lysine residues in transmembrane alpha helices that are critical for LPS binding (Dong et al., 2017; Bertani et al., 2018). Proline can disrupt alpha helices (Richardson, 1981) but it is unlikely that LptG is completely inactivated by proline since it is essential, but it could influence the type of LPS transported, possibly by altering selectivity. One of the more interesting phenotypes associated with the SNP in *lptG* was the observed differences in the transcripts of three genes in E40V. It is unknown how the SNP resulted in differences in the expression or stability of these transcripts but changes in membrane permeability could contribute to the observed difference.

*yciE* is in an operon with catalase *katN* and *yciGF* that contains a putative RpoS-regulated promoter (Robbe-Saule et al., 2001). The *yciGFE-katN* operon is upregulated in response to bile, likely in an RpoS independent manner (Prouty et al., 2004b); however, the greater level of *yciE* detected in these studies was in the absence of bile salts in exponential-phase cells. *yciE* is annotated as a putative DNA damage stress response gene and bile salts can cause DNA damage (Merritt and Donaldson, 2009); therefore, YciE might contribute to bile salts tolerance noted in E40V. The second gene that was expressed at a greater level in E40V and E40E was *yjbE*. This gene is part of the EPS production operon *yjbEFGH* and partially regulated by the RcsCDB phosphorelay pathway. Due to differences in stability, *yjbE* mRNA is often detected in greater amounts than *yjbFGH* mRNAs (Ferrières et al., 2007). The differences in mRNA stability could mean that the entire *yjb* operon was upregulated in E40V and E40E, but only *yjbE* mRNA was detected. Upregulation of this operon likely explains the increase in staining of CBPS in E40V and E40E in comparison to E40. Alternatively, LptG (p.leu26pro) might export atypical LPS that enhances staining of CBPS. Unlike *yciE* and *yjbE*, the gene encoding for ASPP is mostly uncharacterized and is described in NCBI as required for adaptive acid tolerance. Increased expression of ASPP is likely responsible for the observed increase in acid tolerance in E40V and E40E. ASPP expression is in part controlled by RpoN (nitrogen limitation sigma factor, σ^54^) in an indirect manner (Bono et al., 2017).

The regulation of *yciE*, *yjbE*, and ASPP has been partially attributed to RpoS, Rcs phosphorelay, and RpoN, respectively. Changes in the cellular envelope could trigger intracellular stress and RpoS production as well as the Rcs phosphorelay signaling (Majdalani and Gottesman, 2005) that would account for the greater levels of *yciE* or *yjbE*. However, an increase in RpoS or Rcs should result in more extensive transcriptional differences than were observed. Similarly, regulation of ASPP by RpoN would also cause additional transcriptional differences that were not observed (Bono et al., 2017). Interestingly, the regulation of *yciE*, *yjbE*, and ASPP has only been partially attributed to the above-mentioned regulators, and the regulation of *yciE* in response to bile salts is likely independent of RpoS (Prouty et al., 2004b). It is possible that a yet to be described effector could be responsible for the upregulation of these genes in E40V. Although *yciE*, *yjbE*, and ASPP are not positioned close enough in the chromosome to be within the same operon, they may share an undescribed regulatory motif that allows for coregulation. Further elucidation of the relationship between the SNP in *ltpG* and differences in the level of these three transcripts will be a subject of future work.

Engineering the substitution of proline for leucine at LptG position 26 resulted in all of the phenotypes reported for E40V confirming the role of the SNP in the observed phenotypes. While mutations in Lpt pathway proteins that enhance bile salts tolerance can be selected for in experimental systems, there may not be a strong selective pressure for these mutations in hosts or natural environments. During infection, *Salmonella* encounters bile concentrations ranging from 0.2 to 2% in the small intestine to as high as 8% in the gall bladder (Gunn, 2000). However, the E40V variant *lptG* sequence was not identified in the NCBI database that contains a large number of *Salmonella* genomes indicating this form of LptG is not beneficial in natural populations or may even be deleterious.

Clonal and near clonal *Salmonella* populations can contain phenotypically distinct subpopulations, created through a variety of mechanisms including inversions of promoter sequences (Andrewes, 1922; Stocker, 1949; Bonifield and Hughes, 2003; Aldridge et al., 2006; Stewart and Cookson, 2014), SNPs (Piddock et al., 1998; Giraud et al., 1999; Levy et al., 2004; Le et al., 2007; Hernández et al., 2012), and heritable methylation patterns (Broadbent et al., 2010; Cota et al., 2015; Cota and Casadesús, 2016). Next Generation Sequencing (NGS) technologies make genetic and epigenetic (Pirone-Davies et al., 2015) comparisons possible, and these analyses can be combined with traditional bench techniques. While NGS approaches have clear advantages, they can also have limitations. Our assemblies produced using both PacBio and Illumina reads, with a total coverage nearing 300×, contained multiple errors that were rectified by Sanger sequencing. Many of the errors were in genes that had closely related homologs elsewhere in the genome or were in repetitive nucleotide regions. The error rate achieved in our assemblies was likely acceptable for many applications; however, the results from this study demonstrate that a single base pair change can have broad phenotypic effects. Comparative analyses using assembled genomes should take into account the possibility of assembly errors.

*Salmonella enterica* utilizes phenotypic heterogeneity to enhance fitness for a varied hosts and environments. In this study, it was demonstrated that a SNP in the LPS transport gene *lptG* resulted in transcriptional differences in three genes that were linked to identified phenotypes. Additionally, the SNP resulted in an outer membrane that rendered strains E40V and E40E susceptible to novobiocin. Future studies will focus on deciphering the mechanism driving the observed differences in the levels of *yjbE, yciE*, and ASPP transcripts.

## Data Availability Statement

The datasets presented in this study can be found in online repositories. The names of the repository/repositories and accession number(s) can be found below: https://www.ncbi.nlm.nih.gov/genbank/, CP038432; https://www.ncbi.nlm.nih.gov/genbank/, CP038433; https://www.ncbi.nlm.nih.gov/genbank/, CP038434; https://www.ncbi.nlm.nih.gov/genbank/, CP038435.

## Author Contributions

CK and AW developed the concept of the study. ES and TW assembled the DNA sequence data into draft genomes, conducted genome comparisons, and analyzed RNAseq data. TW, JG, and AS performed the experiments and conducted statistical analyses. TW and CK drafted the manuscript. All authors have read and approved the manuscript.

## Conflict of Interest

The authors declare that the research was conducted in the absence of any commercial or financial relationships that could be construed as a potential conflict of interest.

## Supplementary Material

Supplementary Figure 1 Comparison of Xba1 digests of *S. enterica* serovar Typhimurium strains E40 and E40V by pulsed field electrophoresis. A lambda ladder was used as a molecular size marker (kb).

## References

[B1] Aldridge P. D. Wu C. Gnerer J. Karlinsey J. E. Hughes K. T. Sachs M. S. (2006). Regulatory protein that inhibits both synthesis and use of the target protein controls flagellar phase variation in *Salmonella enterica*. *Proc. Natl. Acad. Sci. U.S.A.* 103 11340–11345. 10.1073/pnas.0602127103 16844786 PMC1544088

[B2] Andrewes F. W. (1922). Studies in group agglutination. *J. Pathol.* 25 505–521.

[B3] Aziz R. K. Bartels D. Best A. DeJongh M. Disz T. Edwards R. A. (2008). The RAST server: rapid annotations using subsystems technology. *BMC Genomics* 9:75. 10.1186/1471-2164-9-75 18261238 PMC2265698

[B4] Bankevich A. Nurk S. Antipov D. Gurevich A. A. Dvorkin M. Kulikov A. S. (2012). SPAdes: a new genome assembly algorithm and its applications to single-cell sequencing. *J. Comput. Biol.* 19 455–477. 10.1089/cmb.2012.0021 22506599 PMC3342519

[B5] Battesti A. Majdalani N. Gottesman S. (2011). The RpoS-mediated general stress response in *Escherichia coli*. *Annu. Rev. Microbiol.* 65 189–213. 10.1146/annurev-micro-090110-102946 21639793 PMC7356644

[B6] Bäumler A. J. (1997). The record of horizontal gene transfer in *Salmonella*. *Trends Microbiol.* 5 318–322. 10.1016/S0966-842X(97)01082-2 9263410

[B7] Benjamini Y. Hochberg Y. (1995). Controlling the false discovery rate: a practical and powerful approach to multiple testing. *J. Royal Statist. Soc.* 57, 289–300. http://www.jstor.org/stable/2346101}

[B8] Bertani B. R. Taylor R. J. Nagy E. Kahne D. Ruiz N. (2018). A cluster of residues in the lipopolysaccharide exporter that selects substrate variants for transport to the outer membrane. *Mol. Microbiol.* 109 541–554. 10.1111/mmi.14059 29995974 PMC6200341

[B9] Bonifield H. R. Hughes K. T. (2003). Flagellar phase variation in *Salmonella enterica* is mediated by a posttranscriptional control mechanism. *J. Bacteriol.* 185 3567–3574. 10.1128/JB.185.12.3567 12775694 PMC156219

[B10] Bono A. C. Hartman C. E. Solaimanpour S. Tong H. Porwollik S. McClelland M. (2017). Novel DNA binding and regulatory activities for σ54(RpoN) in *Salmonella enterica* serovar Typhimurium 14028s. *J. Bacteriol.* 199:e00816-16. 10.1128/JB.00816-16 28373272 PMC5446619

[B11] Brettin T. Davis J. J. Disz T. Edwards R. A. Gerdes S. Olsen G. J. (2015). RASTtk: a modular and extensible implementation of the RAST algorithm for building custom annotation pipelines and annotating batches of genomes. *Sci. Rep.* 5:8365. 10.1038/srep08365 25666585 PMC4322359

[B12] Broadbent S. E. Davies M. R. Van Der Woude M. W. (2010). Phase variation controls expression of *Salmonella* lipopolysaccharide modification genes by a DNA methylation-dependent mechanism. *Mol. Microbiol.* 77 337–353. 10.1111/j.1365-2958.2010.07203.x 20487280 PMC2909390

[B13] Buchrieser C. Brosch R. Buchrieser O. Kristl A. Luchansky J. B. Kaspar C. W. (1997). Genomic analyses of *Salmonella enteritidis* phage type 4 strains from Austria and phage type 8 strains from the United States. *Zentralbl. Bakteriol.* 285 379–388. 10.1016/S0934-8840(97)80004-7 9084111

[B14] Chart H. Ward L. R. Rowe B. (1991). Expression of lipopolysaccharide by phage types of *Salmonella enteritidis*. *Lett. Appl. Microbiol.* 13 39–41. 10.1111/j.1472-765X.1991.tb00564.x

[B15] Chimalakonda G. Ruiz N. Chng S. Garner R. A. Kahne D. (2011). Lipoprotein LptE is required for the assembly of LptD by the β-barrel assembly machine in the outer membrane of *Escherichia coli*. *Proc. Natl. Acad. Sci. U.S.A.* 108 2492–2497. 10.1073/pnas.1019089108 21257909 PMC3038771

[B16] Cota I. Casadesús J. (2016). “Formation of bacterial lineages in *Salmonella enterica* by epigenetic mechanisms,” in *Epigenetics–A Different Way of Looking at Genetics. Epigenetics and Human Health*, eds Doerfler W. Böhm P. (Cham: Springer).

[B17] Cota I. Sánchez-romero M. A. Hernández S. B. Pucciarelli M. G. García-del Portillo F. Casadesús J. (2015). Epigenetic control of *Salmonella enterica* O- antigen chain length: a tradeoff between virulence and bacteriophage resistance. *PLoS Genet.* 11:e1005667. 10.1371/journal.pgen.1005667 26583926 PMC4652898

[B18] Crawford R. W. Rosales-reyes R. Luz R.-A. M. de la Chapa-Azuela O. Alpuche-Aranda C. Gunn J. S. (2010). Gallstones play a significant role in *Salmonella* spp. gallbladder colonization and carriage. *Proc. Natl. Acad. Sci. U.S.A.* 107 4353–4358. 10.1073/pnas.1000862107 20176950 PMC2840110

[B19] Crump J. A. Luby S. P. Mintz E. D. (2004). The global burden of typhoid fever. *Bull. World Health Organ.* 82 346–353. 10.1590/S0042-96862004000500008 15298225 PMC2622843

[B20] Darling A. E. Mau B. Perna N. T. (2010). ProgressiveMauve: multiple genome alignment with gene gain, loss and rearrangement. *PLoS One* 5:e11147. 10.1371/journal.pone.0011147 20593022 PMC2892488

[B21] Davis M. R. Jr. Goldberg J. B. (2012). Purification and visualization of lipopolysaccharide from Gram-negative bacteria by hot aqueous-phenol extraction. *J. Vis. Exp* 63:e3916. 10.3791/3916 22688346 PMC3466933

[B22] Dechet A. M. Herman K. M. Chen Parker C. Taormina P. Johanson J. Tauxe R. V. (2014). Outbreaks caused by sprouts, United States, 1998-2010: lessons learned and solutions needed. *Foodborne Pathog. Dis.* 11 635–644. 10.1089/fpd.2013.1705 25076040

[B23] Delcour A. H. (2010). Outer membrane permeability and antibiotic resistance. *Biochim. Biophys. Acta* 1794 808–816. 10.1016/j.bbapap.2008.11.005.Outer PMC2696358 19100346

[B24] Dong H. Xiang Q. Gu Y. Wang Z. Paterson N. G. Stansfeld P. J. (2014). Structural basis for outer membrane lipopolysaccharide insertion. *Nature* 511 52–56. 10.1038/nature13464 24990744

[B25] Dong H. Zhang Z. Tang X. Paterson N. G. Dong C. (2017). Structural and functional insights into the lipopolysaccharide ABC transporter LptB2FG. *Nat. Commun.* 8:222. 10.1038/s41467-017-00273-5 28790314 PMC5548808

[B26] Edgar R. C. (2004). MUSCLE: multiple sequence alignment with high accuracy and high throughput. *Nucleic Acids Res.* 32 1792–1797. 10.1093/nar/gkh340 15034147 PMC390337

[B27] Ferrières L. Aslam S. N. Cooper R. M. Clarke D. J. (2007). The *yjbEFGH* locus in *Escherichia coli* K-12 is an operon encoding proteins involved in exopolysaccharide production. *Microbiology* 153 1070–1080. 10.1099/mic.0.2006/002907-0 17379715

[B28] Flemming H. (2016). EPS—then and now. *Microorganisms* 4:41. 10.3390/microorganisms4040041 27869702 PMC5192524

[B29] Flemming H. C. Wingender J. (2010). The biofilm matrix. *Nat. Rev. Microbiol.* 8 623–633. 10.1038/nrmicro2415 20676145

[B30] Foster J. W. Hall H. K. (1990). Adaptive acidification tolerance response of *Salmonella typhimurium*. *J. Bacteriol.* 172 771–778. 10.1128/jb.172.2.771-778.1990 2404956 PMC208505

[B31] Freudenberg M. A. Tchaptchet S. Keck S. Fejer G. Huber M. Schutze N. (2008). Lipopolysaccharide sensing an important factor in the innate immune response to Gram-negative bacterial infections: benefits and hazards of LPS hypersensitivity. *Immunobiology* 213 193–203. 10.1016/j.imbio.2007.11.008 18406367

[B32] Giraud E. Brisabois A. Martel J. L. Chaslus-Dancla E. (1999). Comparative studies of mutations in animal isolates and experimental in vitro- and in vivo-selected mutants of *Salmonella* spp. suggest a counterselection of highly fluoroquinolone-resistant strains in the field. *Antimicrob. Agents Chemother.* 43 2131–2137. 10471553 10.1128/aac.43.9.2131 PMC89435

[B33] Gonzalez-Escobedo G. Gunn J. S. (2013). Gallbladder epithelium as a niche for chronic *Salmonella* carriage. *Infect. Immun.* 81 2920–2930. 10.1128/IAI.00258-13 23732169 PMC3719562

[B34] Gonzalez-Escobedo G. Marshall J. M. Gunn J. S. (2011). Chronic and acute infection of the gall bladder by *Salmonella* Typhi: understanding the carrier state. *Nat. Rev. Microbiol.* 9 9–14. 10.1038/nrmicro2490.Chronic 21113180 PMC3255095

[B35] Groisman E. A. Ochman H. (1997). How *Salmonella* became a pathogen. *Trends Microbiol.* 5 343–349. 10.1016/S0966-842X(97)01099-8 9294889

[B36] Gunn J. S. (2000). Mechanisms of bacterial resistance and response to bile. *Microbes Infect.* 2 907–913. 10.1016/S1286-4579(00)00392-0 10962274

[B37] Gunn J. S. Marshall J. M. Baker S. Dongol S. Charles R. C. Ryan E. T. (2014). *Salmonella* chronic carriage: epidemiology, diagnosis, and gallbladder persistence. *Trends Microbiol.* 22 648–655. 10.1016/j.tim.2014.06.007 25065707 PMC4252485

[B38] Guo M. S. Gross C. A. (2014). Stress-induced remodeling of the bacterial proteome. *Curr. Biol.* 24 R424–R434. 10.1016/j.cub.2014.03.023 24845675 PMC4089988

[B39] Hamad M. A. Di Lorenzo F. Molinaro A. Valvano M. A. (2012). Aminoarabinose is essential for lipopolysaccharide export and intrinsic antimicrobial peptide resistance in *Burkholderia cenocepacia*. *Mol. Microbiol.* 85 962–974. 10.1111/j.1365-2958.2012.08154.x 22742453

[B40] Harrison L. B. Fowler R. C. Abdalhamid B. Selmecki A. Hanson N. D. (2019). lptG contributes to changes in membrane permeability and the emergence of multidrug hypersusceptibility in a cystic fibrosis isolate of *Pseudomonas aeruginosa*. *Microbiologyopen* 8:e844. 10.1002/mbo3.844 30977288 PMC6854846

[B41] Hawkins J. P. Geddes B. A. Oresnik I. J. (2017). Common dyes used to determine bacterial polysaccharides on agar are affected by medium acidification. *Can. J. Microbiol.* 63 559–562. 10.1139/cjm-2016-0743 28253454

[B42] Hengge-Aronis R. (2002). Signal transduction and regulatory mechanisms involved in control of the sigma(S) (RpoS) subunit of RNA polymerase. *Microbiol. Mol. Biol. Rev.* 66 373–395. 10.1128/MMBR.66.3.373 12208995 PMC120795

[B43] Hernández S. B. Cota I. Ducret A. Aussel L. Casadesús J. (2012). Adaptation and preadaptation of *Salmonella enterica* to bile. *PLoS Genet.* 8:e1002459. 10.1371/journal.pgen.1002459 22275872 PMC3261920

[B44] Isaacs S. Aramini J. Ciebin B. Farrar A. Ahmed R. Middleton D. (2005). An international outbreak of salmonellosis associated with raw almonds contaminated with a rare phage type of *Salmonella enteriditis*. *J. Food Prot.* 68 191–198. 15690826 10.4315/0362-028x-68.1.191

[B45] Kalynych S. Morona R. Cygler M. (2014). Progress in understanding the assembly process of bacterial O-antigen. *FEMS Microbiol Rev.* 38 1048–1065. 24617522 10.1111/1574-6976.12070

[B46] Lai C. W. Chan R. C. Y. Cheng A. F. B. Sung J. Y. Leung J. W. C. (1992). Common bile duct stones: a cause of chronic salmonellosis. *Am. J. Gastroenterol.* 87 1198–1199. 1519582

[B47] Lan R. Reeves P. R. Octavia S. (2009). Population structure, origins and evolution of major *Salmonella enterica* clones. *Infect. Genet. Evol.* 9 996–1005. 10.1016/j.meegid.2009.04.011 19393770

[B48] Le T. A. H. Fabre L. Roumagnac P. Grimont P. A. D. Scavizzi M. R. Weill F. X. (2007). Clonal expansion and microevolution of quinolone-resistant *Salmonella enterica* serotype typhi in Vietnam from 1996 to 2004. *J. Clin. Microbiol.* 45 3485–3492. 10.1128/JCM.00948-07 17728470 PMC2168509

[B49] Lesse A. J. Campagnari A. A. Bittner W. E. Apicella M. A. (1990). Increased resolution of lipopolysaccharides and lipooligosaccharides utilizing tricine-sodium dodecyl sulfate-polyacrylamide gel electrophoresis. *J. Immunol. Methods* 126 109–117. 10.1016/0022-1759(90)90018-Q 2106001

[B50] Levy D. D. Sharma B. Cebula T. A. (2004). Single-nucleotide polymorphism mutation spectra and resistance to quinolones in *Salmonella enterica* serovar Enteritidis with a mutator phenotype. *Antimicrob. Agents Chemother.* 48 2355–2363. 10.1128/AAC.48.7.2355-2363.2004 15215081 PMC434170

[B51] Liao Y. Smyth G. K. Shi W. (2013). The Subread aligner: fast, accurate and scalable read mapping by seed-and-vote. *Nucleic Acids Res.* 41:e108. 10.1093/nar/gkt214 23558742 PMC3664803

[B52] Love M. I. Huber W. Anders S. (2014). Moderated estimation of fold change and dispersion for RNA-seq data with DESeq2. *Genome Biol.* 15:550. 10.1186/s13059-014-0550-8 25516281 PMC4302049

[B53] Luo Q. Yang X. Yu S. Shi H. Wang K. Xiao L. (2017). Structural basis for lipopolysaccharide extraction by ABC transporter LptB2FG. *Nat. Struct. Mol. Biol.* 24 469–474. 10.1038/nsmb.3399 28394325

[B54] Madden T. (2002). “The BLAST sequence analysis tool,” in *The NCBI Handbook (Internet)*, eds McEntyre J. Ostell J. (Bethesda, MD: National Center for Biotechnology Information). Available online at: https://www.ncbi.nlm.nih.gov/books/NBK21097/

[B55] Mahon B. E. Pönkä A. Hall W. N. Komatsu K. Dietrich S. E. Siitonen A. (1997). An international outbreak of *Salmonella* infections caused by *Alfalfa sprouts* grown from contaminated seeds. *J. Infect. Dis.* 175 876–882. 10.1086/513985 9086144

[B56] Majdalani N. Gottesman S. (2005). The RCS phosphorelay: a complex signal transduction system. *Annu. Rev. Microbiol.* 59 379–405. 10.1146/annurev.micro.59.050405.101230 16153174

[B57] Majowicz S. E. Musto J. Scallan E. Angulo F. J. Kirk M. O’Brien S. J. (2010). The global burden of nontyphoidal *Salmonella gastroenteritis*. *Clin. Infect. Dis.* 50 882–889. 10.1086/650733 20158401

[B58] Mellefont L. A. McMeekin T. A. Ross T. (2005). Viable count estimates of lag time responses for *Salmonella typhimurium* M48 subjected to abrupt osmotic shifts. *Int. J. Food Microbiol.* 105 399–410. 10.1016/ijfoodmicro.2005.03.018 16109449

[B59] Merritt M. E. Donaldson J. R. (2009). Effect of bile salts on the DNA and membrane integrity of enteric bacteria. *J. Med. Microbiol.* 58 1533–1541. 10.1099/jmm.0.014092-0 19762477

[B60] Narita S. Tokuda H. (2009). Biochemical characterization of an ABC transporter LptBFGC complex required for the outer membrane sorting of lipopolysaccharides. *FEBS Lett.* 583 2160–2164. 10.1016/j.febslet.2009.05.051 19500581

[B61] Newell D. G. Koopmans M. Verhoef L. Duizer E. Aidara-Kane A. Sprong H. (2010). Food-borne diseases–The challenges of 20 years ago still persist while new ones continue to emerge. *Int. J. Food Microbiol.* 139 S3–S15. 10.1016/j.ijfoodmicro.2010.01.021 20153070 PMC7132498

[B62] Nikaido E. Yamaguchi A. Nishino K. (2008). AcrAB multidrug efflux pump regulation in *Salmonella enterica* serovar Typhimurium by RamA in response to environmental signals. *J. Biol. Chem.* 283 24245–24253. 10.1074/jbc.M804544200 18577510 PMC2527123

[B63] Nikaido H. (2003). Molecular basis of bacterial outer membrane permeability revisited. *Microbiol. Mol. Biol. Rev.* 67 593–656. 10.1128/MMBR.67.4.593 14665678 PMC309051

[B64] Nikolenko S. I. Korobeynikov A. I. Alekseyev M. A. (2013). BayesHammer: bayesian clustering for error correction in single-cell sequencing. *BMC Genomics* 14:S7. 10.1186/1471-2164-14-S1-S7 23368723 PMC3549815

[B65] Nobre T. M. Martynowycz M. W. Andreev K. Kusmenk I. Nikaido H. Gidalevitz D. (2015). Modification of *Salmonella* lipopolysaccharides prevents outer membrane penetration of Novobiocin. *Biophys. J.* 109 2537–2545. 10.1016/j.bpj.2015.10.013 26682812 PMC4699856

[B66] Okuda S. Sherman D. J. Silhavy T. J. Ruiz N. Kahne D. (2016). Lipopolysaccharide transport and assembly at the outer membrane: the PEZ model. *Nat. Rev. Microbiol.* 14 337–345. 10.1038/nrmicro.2016.25.Lipopolysaccharide 27026255 PMC4937791

[B67] Overbeek R. Olson R. Pusch G. D. Olsen G. J. Davis J. J. Disz T. (2014). The SEED and the rapid annotation of microbial genomes using Subsystems Technology (RAST). *Nucleic Acids Res.* 42 206–214. 10.1093/nar/gkt1226 24293654 PMC3965101

[B68] Painter J. A. Hoekstra R. M. Ayers T. Tauxe R. V. Braden C. R. Angulo F. J. (2013). Attribution of foodborne illnesses, hospitalizations, and deaths to food commodities by using outbreak data, United States, 1998-2008. *Emerg. Infect. Dis.* 19 407–415. 23622497 10.3201/eid1903.111866 PMC3647642

[B69] Pfaffl M. W. (2001). A new mathematical model for relative quantification in real-time RT-PCR. *Nucleic Acids Res.* 29:e45. 10.1093/nar/29.9.e45 11328886 PMC55695

[B70] Piddock L. J. V. Ricci V. Mclaren I. Griggs D. J. (1998). Role of mutation in the *gyrA* and *parC* genes of nalidixic-acid-resistant *Salmonella* serotypes from animals in the United Kingdom. *J. Antimicrob. Chemother.* 41 635–641. 9687102 10.1093/jac/41.6.635

[B71] Pirone-Davies C. Hoffmann M. Roberts R. J. Muruvanda T. Timme R. E. Strain E. (2015). Genome-wide methylation patterns in *Salmonella enterica* subsp. enterica Serovars. *PLoS One* 10:e0123639. 10.1371/journal.pone.0123639 25860355 PMC4393132

[B72] Prouty A. M. Brodsky I. E. Falkow S. Gunn J. S. (2004a). Bile-salt-mediated induction of antimicrobial and bile resistance in *Salmonella typhimurium*. *Microbiology* 150 775–783. 10.1099/mic.0.26769-0 15073288

[B73] Prouty A. M. Brodsky I. E. Manos J. Belas R. Falkow S. Gunn J. S. (2004b). Transcriptional regulation of *Salmonella enterica* serovar Typhimurium genes by bile. *FEMS Immunol. Med. Microbiol.* 41 177–185. 10.1016/j.femsim.2004.03.002 15145463

[B74] Reisch C. R. Prather K. L. J. (2015). The no-SCAR (Scarless Cas9 Assisted Recombineering) system for genome editing in *Escherichia coli*. *Sci. Rep.* 5:15096. 10.1038/srep15096 26463009 PMC4604488

[B75] Reisch C. R. Prather K. L. J. (2017). Scarless Cas9 assisted recombineering (no-SCAR) in *Escherichia coli*, an easy-to-use system for genome editing. *Curr. Protoc. Mol. Biol.* 2017 1–20. 10.1002/cpmb.29 28060411

[B76] Richardson J. S. (1981). The anatomy and taxonomy of protein structure. *Adv. Prot. Chem.* 34 167–339. 10.1016/s0065-3233(08)60520-3 7020376

[B77] Richter A. M. Povolotsky T. L. Wieler L. H. Hengge R. (2014). Cyclic-di-GMP signalling and biofilm-related properties of the Shiga toxin-producing 2011 German outbreak *Escherichia coli* O104:H4. *EMBO Mol. Med.* 6 1622–1637. 10.15252/emmm.201404309 25361688 PMC4287979

[B78] Rio D. C. Ares M. Hannon G. J. Nilsen T. W. (2010). Purification of RNA using TRIzol (TRI Reagent). *Cold Spring Harb. Protoc.* 2010:db.prot5439. 10.1101/pdb.prot5439 20516177

[B79] Robbe-Saule V. Coynault C. Ibanez-Ruiz M. Hermant D. Norel F. (2001). Identification of a non-haem catalase in *Salmonella* and its regulation by RpoS (σs). *Mol. Microbiol.* 39 1533–1545. 10.1046/j.1365-2958.2001.02340.x 11260470

[B80] Robinson J. T. Thorvaldsdottir H. Winckler W. Guttman M. Lander E. S. Getz G. (2012). Integrative genomics viewer. *Nat. Biotechnol.* 29 24–26. 10.1038/nbt.1754.Integrative PMC3346182 21221095

[B81] Roncarati D. Scarlato V. (2017). Regulation of heat-shock genes in bacteria: from signal sensing to gene expression output. *FEMS Microbiol. Rev.* 41 549–574. 10.1093/femsre/fux015 28402413

[B82] Ruiz N. Gronenberg L. S. Kahne D. Silhavy T. J. (2008). Identification of two inner-membrane proteins required for the transport of lipopolysaccharide to the outer membrane of *Escherichia coli*. *Proc. Natl. Acad. Sci. U.S.A.* 105 5537–5542. 18375759 10.1073/pnas.0801196105 PMC2291135

[B83] Sanger F. Nicklen S. Coulson R. (1977). DNA sequencing with chain-terminating inhibitors. *Proc. Natl. Acad. Sci. U.S.A.* 74 5463–5467. 10.1073/pnas.74.12.5463 271968 PMC431765

[B84] Schindelin J. Arganda-carreras I. Frise E. Kaynig V. Longair M. Pietzsch T. (2012). Fiji: an open-source platform for biological-image analysis. *Nat. Methods* 9 676–682. 10.1038/nmeth.2019 22743772 PMC3855844

[B85] Schneider C. A. Rasband W. S. Eliceiri K. W. (2012). NIH Image to ImageJ: 25 years of image analysis. *Nat. Methods* 9 671–675. 10.1038/nmeth.2089 22930834 PMC5554542

[B86] Sheng H. Lin J. Y. Watkins M. K. Miinich S. A. Hovde C. J. (2008). Characterization of an *Escherichia coli* O157:H7 O-antigen deletion mutant and effect of the deletion on bacterial persistence in the mouse intestine and colonization of the bovine terminal rectal mucosa. *Appl. Environ. Microbiol.* 74 5015–5022. 10.1128/AEM.00743-08.0 18552194 PMC2519267

[B87] Sherman D. J. Xie R. Taylor R. J. George A. H. Okuda S. Foster P. J. (2018). Lipopolysaccharide is transported to the cell surface by a membrane-to-membrane protein bridge. *Science* 359 798–801. 10.1126/science.aar1886.Lipopolysaccharide 29449493 PMC5858563

[B88] Spector M. P. Kenyon W. J. (2012). Resistance and survival strategies of *Salmonella enterica* to environmental stresses. *Food Res. Int.* 45 455–481. 10.1016/j.foodres.2011.06.056

[B89] Sperandeo P. Cescutti R. Villa R. Di Benedetto C. Candia D. Dehò G. (2007). Characterization of *lptA* and *lptB*, two essential genes implicated in lipopolysaccharide transport to the outer membrane of *Escherichia coli*. *J. Bacteriol.* 189 244–253. 10.1128/JB.01126-06 17056748 PMC1797204

[B90] Stewart M. K. Cookson B. T. (2014). Mutually repressing repressor functions and multi-layered cellular heterogeneity regulate the bistable *Salmonella* FliC census. *Mol. Microbiol.* 94 1272–1284. 10.1111/mmi.12828.Mutually 25315056 PMC4262692

[B91] Stocker B. A. D. (1949). Measurements of rate of mutation of flagellar antigenic phase in *Salmonella typhimurium*. *J. Hyg. (Lond).* 47 398–413. 10.1017/s002217240001473x PMC2234939 20475789

[B92] Tamplin M. L. Jackson J. K. Buchrieser C. Murphree R. L. Portier K. M. Gangar V. (1996). Pulsed-field gel electrophoresis and ribotype profiles of clinical and environmental *Vibrio vulnificus* isolates. *Appl. Environ. Microbiol.* 62 3572–3580. 10.1016/j.gexplo.2006.08.011 8837412 PMC168162

[B93] Thornton B. Basu C. (2011). Real-time PCR (qPCR) primer design using free online software. *Biochem. Mol. Biol. Educ.* 39 145–154. 10.1002/bmb.20461 21445907

[B94] Threlfall E. J. (2002). Antimicrobial drug resistance in *Salmonella*: problems and perspectives in food- and water-borne infections. *FEMS Microbiol. Rev.* 26 141–148. 10.1111/j.1574-6976.2002.tb00606.x 12069879

[B95] Untergasser A. Cutcutache I. Koressaar T. Ye J. Faircloth B. C. Remm M. (2012). Primer3-new capabilities and interfaces. *Nucleic Acids Res.* 40:e115. 10.1093/nar/gks596 22730293 PMC3424584

[B96] Walker B. J. Abeel T. Shea T. Priest M. Abouelliel A. Sakthikumar S. (2014). Pilon: an integrated tool for comprehensive microbial variant detection and genome assembly improvement. *PLoS One* 9:e112963. 10.1371/journal.pone.0112963 25409509 PMC4237348

[B97] Whitfield C. Trent M. S. (2014). Biosynthesis and export of bacterial lipopolysaccharides. *Annu. Rev. Biochem.* 83 99–128. 10.1146/annurev-biochem-060713-035600 24580642

[B98] William S. Feil H. Copeland A. (2004). *Bacterial Genomic DNA Isolation Using CTAB.* Walnut Creek, CA: JGI Protoc.

[B99] Zhang G. Meredith T. C. Kahne D. (2013). On the essentiality of lipopolysaccharide to Gram-negative bacteria. *Curr. Opin. Microbiol.* 16 779–785. 10.1016/j.mib.2013.09.007 24148302 PMC3974409

