# Supplementary figures and images for: A Single Nucleotide Polymorphism in lptG Increases Tolerance to Bile Salts, Acid, and Staining of Calcofluor-Binding Polysaccharides in Salmonella enterica Serovar Typhimurium E40
Source: Front Microbiol. 2021 Jun 2;12:671453. doi: 10.3389/fmicb.2021.671453 (PMC8208086; doi:10.3389/fmicb.2021.671453)

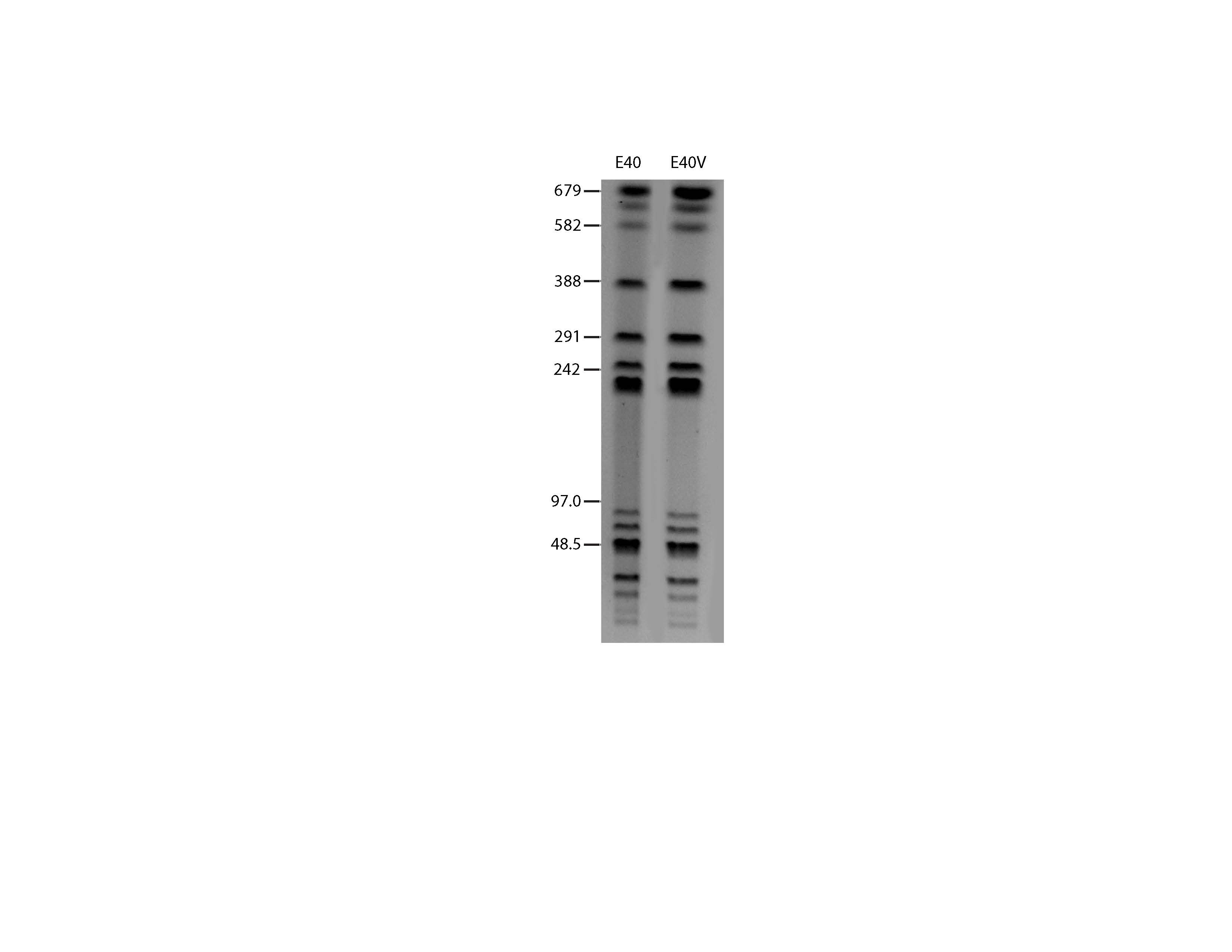

Supplement: Supplementary Figure 1 — Comparison of Xba1 digests of S. enterica serovar Typhimurium strains E40 and E40V by pulsed field electrophoresis. A lambda ladder was used as a molecular size marker (kb). [file Image_1.JPEG]
