# Supplementary material for: A Single Nucleotide Polymorphism in lptG Increases Tolerance to Bile Salts, Acid, and Staining of Calcofluor-Binding Polysaccharides in Salmonella enterica Serovar Typhimurium E40
Source: Front Microbiol. 2021 Jun 2;12:671453. doi: 10.3389/fmicb.2021.671453 (PMC8208086; doi:10.3389/fmicb.2021.671453)
Supplement: Supplementary file 2 [file Table_1.pdf]

TABLE S1 | Primers used in this study

Primer Name	Description	Sequence
TW080 SNP 1 5'	noncoding region sequencing	GTGATGCATTCCGTCTCCTT
TW081 SNP 1 3'	noncoding region sequencing	TTTCCGCCATTCTGGTGTAT
TW082 SNP 2 5'	exodeoxyribonuclease amplification	CAGGGCATCAATAGCTGGTT
TW083 SNP 2 3'	exodeoxyribonuclease amplification	CAGGAAAAGACACCGGACAT
TW084 SNP 2 SEQ 5'	exodeoxyribonuclease sequencing	AATAGCCGATACACGGTTGC
TW085 SNP 2 SEQ 3'	exodeoxyribonuclease sequencing	ATACCGAAACAGGCGAAGTG
TW086 SNP 3 5'	lptG sequencing	CCGTTTTCTGCGTAAAGGAG
TW087 SNP 3 3'	lptG sequencing	ATAGTTACGCGCCATCTGCT
TW088 SNP 4 5'	rcnA sequencing	CGGCTTTTATCATTGCCATT
TW089 SNP 4 3'	rcnA sequencing	GCCGAATAGCAGGATCTGTC
TW098 SNP 5 Redesign F	putative heme lyase subunit amplification	GGGAAAGATGGCAAATCGTA
TW099 SNP 5 Redesign R	putative heme lyase subunit amplification	ATATGCGCGGGTAAACAGTC
TW092 SNP 5 SEQ 5'	putative heme lyase subunit sequence	GAAATGCCAGAACAACAGCA
TW093 SNP 5 SEQ 3'	putative heme lyase subunit sequence	CCATCTCCTCCTCATTCAGC
TW094 SNP 6 5'	phage head-like protein	CCTCCGGACGTTAAATACGA
TW095 SNP 6 3'	phage head-like protein	CATGCCTGGTATGACCACAG
TW096 SNP 7 5'	conjugative transfer protein	GGAAATAACCGGCTGAACAA
TW097 SNP 7 3'	conjugative transfer protein	CAGTGGATTGTGACGGTACG
TW120 recA-F	qPCR primer	GGTCAACCAGTTCGCCATAG
TW121 recA-R	qPCR primer	AATGTCGTGGGTAGCGAAAC
TW122 rpoB-F	qPCR primer	ACGTGCTCTGGAAATCGAAG
TW123 rpoB-R	qPCR primer	CACGGATACGGCTAAACAGG
TW170 YjbE-1/2 F	qPCR primer	CGTAAGTTCGGCGGTGGG
TW171 YjbE-1 R	qPCR primer	TACTGGTGGTCGTGGTGGTC
TW175 YciE-1 F	qPCR primer	AATTGAGGCAGTGTCGCCAG
TW176 YciE-2 F	qPCR primer	TCGGAATTGAGGCAGTGTCG
TW180 acid-2 F	qPCR primer	CTCTGGTTGTTGCCGCTG
TW181 acid 2 R	qPCR primer	TTGTGGTGCTGGGTGGTC
TW199 sgRNA R Primer	no-scar primers	GCATGAACAACGTCATCATGgtgctcagtatctctatcactga
TW200 permease v1	no-scar primers	C*C*A*T*TTTTACCACCATCATGATGACGTTGTTCATGCCAGTGTCGCTCTCCGGGATCATCAAGTTTGTCGATCAGCTGAAA
TW202 Primer CPEC2 F	no-scar primers	CGGCGTCACACTTTGCTAT
TW203 Primer gam R	no-scar primers	TTTATAACCTCCTTAGAGCTCGA
TW204 pKDseq5	no-scar primers	CAGTGAATGGGGGTAAATGG
TW205 sgrnaR	no-scar primers	GCCTGCAGTCTAGACTCGAG
TW206 sgrnaA	no-scar primers	AGCTTTCGCTAAGGATGATTT
TW232 sgRNA F V2	no-scar primers	CATGATGACGTTGTTCATGC GTTTTAGAGCTAGAAATAGCAAG

*indicate phosphorothioate bonds
