# Supplementary material for: A Single Nucleotide Polymorphism in lptG Increases Tolerance to Bile Salts, Acid, and Staining of Calcofluor-Binding Polysaccharides in Salmonella enterica Serovar Typhimurium E40
Source: Front Microbiol. 2021 Jun 2;12:671453. doi: 10.3389/fmicb.2021.671453 (PMC8208086; doi:10.3389/fmicb.2021.671453)
Supplement: Supplementary file 3 [file Table_2.pdf]

TABLE S2 RNA Sequencing Alignment Statistics

	E40			E40V		
	1	2	3	1	2	3
Rsubread Statistics						
Total Reads	16,092,526	16,466,727	15,310,005	17,655,833	11,774,936	16,201,886
Mapped Reads[a]	15,578,741	15,878,663	14,759,350	16,608,480	11,258,242	14,362,812
Percent Mapped	96.8%	96.4%	96.4%	94.1%	95.6%	88.6%
DESeq2 Statistics						
Percent of Reads Assigned[b]	69.9%	69.2%	71.3%	68.8%	69.8%	64.4%

[a] Number of reads mapped to E40 genome using Rsubread
[b] Percent of mapped reads within an coding sequence utilizing E40 RAST annotation
