# Supplementary material for: A Single Nucleotide Polymorphism in lptG Increases Tolerance to Bile Salts, Acid, and Staining of Calcofluor-Binding Polysaccharides in Salmonella enterica Serovar Typhimurium E40
Source: Front Microbiol. 2021 Jun 2;12:671453. doi: 10.3389/fmicb.2021.671453 (PMC8208086; doi:10.3389/fmicb.2021.671453)
Supplement: Supplementary file 5 [file Table_4.docx]

|  | **Strain** | **Raw reads** | **Corrected**  **reads^a^** | **Unpaired**  **reads** | **Total raw**  **sequence (bp)** | **Coverage** |
| --- | --- | --- | --- | --- | --- | --- |
| **A Illumina sequencing statistics (250 bp paired end)** | | | | | |  |
|  | E40 | 1,131,473 | 1,110,510 | 20,918 | 5.68 x 10^8^ | 114x |
|  | E40V | 1,120,701 | 1,102,195 | 18,468 | 5.63 x 10^8^ | 113 x |
|  |  |  |  |  |  |  |
| **B** | **PacBio SMRT sequencing statistics** | | | | |  |
|  | E40 | 115,539 | 8020 |  | 9.27 x 10^8^ | 189 x |
|  | E40V | 90,155 | 8908 |  | 8.03 x 10^8^ | 164 x |
|  | **Strain** | **Contig** | **Contig length (bp)** | **Identity** |  |  |
| **C** | **Assembly statistics** | |  |  |  |  |
|  | E40 | 1 | 4,890,368 | chromosome |  |  |
|  |  | 2 | 94,009 | plasmid |  |  |
|  | E40V | 1 | 4,890,338 | chromosome |  |  |
|  |  | 2 | 94,009 | plasmid |  |  |

**TABLE S4 |**Data from Illumina sequencing, PacBio SMRT sequencing, and assembly of strains E40 and E40V
