# Supplementary material for: A Single Nucleotide Polymorphism in lptG Increases Tolerance to Bile Salts, Acid, and Staining of Calcofluor-Binding Polysaccharides in Salmonella enterica Serovar Typhimurium E40
Source: Front Microbiol. 2021 Jun 2;12:671453. doi: 10.3389/fmicb.2021.671453 (PMC8208086; doi:10.3389/fmicb.2021.671453)
Supplement: Supplementary file 6 [file Table_5.docx]

**TABLE S5 |** Identified chromosome polymorphisms between strains E40 and E40V following DNA sequencing and assembly. Genome assemblies were compared using Mauve, BLAST, and IGV. Putative differences were examined by Sanger sequencing

| **Location** | **Annotation** | **Type** | **Sanger result^a^** |
| --- | --- | --- | --- |
| 552,929 | noncoding region | 1 base gap | Assembly error^b^ |
| 1,102,892-1,103,305 | exodeoxyribonuclease | 3 SNPs^c^ | Assembly error |
| 1,742,628 | Heme lyase, *ccmH-1* | 1 codon deletion | Assembly error |
| 2,830,845-2,830,923 | Prophage head-tail preconnector | 3 SNPs | Assembly error |
| 3,231,413 | Nickel/cobalt efflux, *rcnA* | 4 codon deletion | Assembly error |
| 7,763,350 | LPS transport, *lptG* | 1 SNP | Confirmed SNP |

^a^Regions containing putative differences were amplified by PCR and sequenced by Sanger sequencing

^b^Assembly error, putative difference not confirmed by Sanger sequencing

^c^SNP, single nucleotide polymorphism
